# Ten-Year Trends in Candidemia at a Tertiary-Care Hospital in Spain (2015–2024): Epidemiological Shifts, Diagnostic Acceleration, and Impact of Antifungal Stewardship in the COVID-19 and Post-Pandemic Era

**DOI:** 10.3390/jof12060428

**Published:** 2026-06-11

**Authors:** Cristian Castelló-Abietar, Miguel Alaguero, Enrique García-Carús, Emilio García-Prieto, Silvia Bolaños, Jonathan Fernández-Suárez, Teresa Peláez García de la Rasilla

**Affiliations:** 1 Microbiology Department, Hospital Universitario Central de Asturias, 33001 Oviedo, Spain; silviabolanosgarcia@gmail.com (S.B.); jhonatan.fernandez@sespa.es (J.F.-S.); 2 Microbiology and Infectious Diseases Group, Instituto de Investigación Sanitaria del Principado de Asturias (ISPA), 33001 Oviedo, Spain; 3 Clinical Pharmacy Management Department, Hospital Universitario Central de Asturias, 33011 Oviedo, Spain; miguel.alaguero@sespa.es; 4 Department of Internal Medicine, Hospital Universitario Central de Asturias, 33011 Oviedo, Spain; e.g.carus@gmail.com; 5 Intensive Care Unit, Hospital Universitario Central de Asturias, 33011 Oviedo, Spain; egarciaprieto@gmail.com; 6 Grupo Investigación Traslacional en el Paciente Crítico, Instituto de Investigación Sanitaria del Principado de Asturias (ISPA), 33011 Oviedo, Spain; 7 CIBER of Respiratory Diseases (CIBERES), ISCIII, 28029 Madrid, Spain

**Keywords:** candidemia, fungal etiology, risk factors, diagnostic turnaround time, multiplex PCR, antifungal resistance, antifungal stewardship, COVID-19, ICU, mortality

## Abstract

Candidemia is a major healthcare-associated bloodstream infection with high mortality, requiring ongoing surveillance to guide management. This retrospective study analyzed 306 candidemia episodes diagnosed between 2015 and 2024 at a Spanish tertiary-care hospital, comparing two periods (2015–2019 vs. 2020–2024). The overall incidence was 0.79 episodes per 1.000 admissions, with peaks in 2021 and 2024. *Candida albicans* was the most common species (44.8%), followed by *Candida parapsilosis* (19.0%) and *Nakaseomyces glabrata* (15.7%). A significant epidemiological shift occurred in the later period, with increased *C. albicans*, decreased *C. parapsilosis*, and emergence of *N. glabrata* as the second-most frequent species. ICU-related cases rose significantly during the COVID-19 period. Diagnostic turnaround times improved, including faster blood culture positivity and species identification by MALDI-TOF, supported by rapid PCR testing with high sensitivity (91.7%). Antifungal resistance to fluconazole was notable in *N. glabrata* (48.1%). Empirical echinocandin use increased, alongside greater targeted fluconazole therapy. Antimicrobial stewardship interventions, mainly de-escalation strategies, were widely implemented after 2019. Overall mortality was 40.8%, with a decline observed in 2023–2024. These findings suggest that integrated diagnostic and stewardship strategies may improve outcomes, though causal relationships require further study.

## 1. Introduction

Candidemia is a major cause of healthcare-associated bloodstream infection and represents the most frequent manifestation of invasive candidiasis [1]. The incidence of candidemia ranges between 2 and 14 cases per 100,000 population, according to population-based studies [2,3].

The burden of candidemia is further amplified by prolonged hospitalization, increased healthcare costs, and the need for complex diagnostic and therapeutic strategies. Despite advances in antifungal therapy and supportive care, mortality remains high, often exceeding 30–40%, particularly in critically ill patients [4,5].

The epidemiology of candidemia is dynamic and varies considerably across geographic regions, healthcare settings, and patient populations. These differences are driven by a complex interplay of factors, including patient demographics, underlying comorbidities, immunosuppressive conditions, exposure to invasive procedures, antimicrobial pressure, and local infection prevention and control practices. Although *Candida albicans* remains the leading cause of candidemia worldwide, a progressive shift toward non-albicans *Candida* species has been reported in many centers over the last two decades. This trend is clinically significant because different species exhibit distinct virulence characteristics, antifungal susceptibility profiles, and resistance mechanisms, which may directly influence therapeutic decisions and patient outcomes [6].

Among non-albicans *Candida* species, *Candida parapsilosis* has particular epidemiological relevance in several European countries, especially in Mediterranean regions, where it frequently ranks among the most common causes of candidemia. This species is strongly associated with intravascular devices, catheter-related bloodstream infections, parenteral nutrition, and healthcare-associated transmission, making it a frequent cause of hospital outbreaks. Furthermore, the emergence of isolates with reduced susceptibility or resistance to azoles, together with its intrinsically higher echinocandin MICs compared with other *Candida* species, has raised important concerns regarding empirical antifungal selection and step-down treatment strategies [7].

Among these, *Nakaseomyces glabrata* has emerged as a key pathogen due to its reduced susceptibility to azoles and its capacity to develop resistance during treatment. Similarly, intrinsically resistant species such as *Pichia kudriavzevii* pose significant therapeutic challenges. Consequently, continuous local surveillance of species distribution and antifungal susceptibility patterns is essential to guide empirical therapy, optimize antifungal stewardship interventions, and detect emerging epidemiological trends at an early stage [8,9].

Moreover, recent temperature changes associated with global warming have been proposed as an ecological factor capable of modifying fungal population dynamics, as seen with the near-simultaneous emergence of distinct clades of *Candidozyma auris* (*Candida auris*) as nosocomial pathogens across several continents [9,10].

The COVID-19 pandemic introduced substantial changes in healthcare systems, including increased ICU admissions, widespread use of immunomodulatory therapies, and prolonged exposure to invasive devices. These factors have been associated with an increased incidence of invasive fungal infections among hospitalized patients. In Europe, the most frequently observed were candidiasis and aspergillosis, while mucormycosis predominated in India [11]. *C. albicans* was the most common yeast species in critically ill COVID-19 patients (44% of candidemia cases in a U.S. multicenter study), although in some regions the emerging pathogen *C. auris* predominated [12].

In parallel, advances in diagnostic microbiology have improved the speed and accuracy of pathogen identification, while blood culture remains the gold standard [5,13] because it is the only method that allows isolation, full identification, and preservation of the causative pathogen as well as subsequent antifungal susceptibility testing [14]. However, the sensitivity of blood cultures in invasive candidiasis is suboptimal, with a high rate of false negatives [14,15]. This limitation stems from the need for viable cells for detection. The average *Candida* concentration in an initial positive blood culture is approximately 1 colony-forming unit (CFU)/mL, corresponding to about 5.6 × 10^3^ CFU in an adult’s total blood volume. Since a 10 mL culture sample represents only 0.2% of systemic circulation, and 26–65% of positive cultures contain fewer than 1 CFU/mL, negative cultures may reflect either the absence of viable *Candida* cells, concentrations below the detection limit, or intermittent bloodstream release [16].

Its limitations have driven the adoption of rapid diagnostic technologies such as MALDI-TOF and multiplex PCR panels. These tools enable earlier identification of pathogens and may facilitate timely optimization of antifungal therapy. Key recommendations emphasize combining traditional methods (direct microscopy, culture) with rapid and specific diagnostic tools to enhance diagnostic accuracy [7,17,18,19].

Antifungal stewardship programs have become increasingly important in optimizing antifungal use, emphasizing the integration of rapid diagnostics and stewardship interventions as a combined strategy to improve outcomes [20]. The standard treatment duration of 14 days after the first negative blood culture has been questioned by some studies suggesting that shorter courses may be feasible [21], potentially reducing antifungal exposure and selective pressure—especially relevant given the emergence of multidrug-resistant *Candida* species [22].

The objectives of this study were to analyze temporal trends in candidemia epidemiology, evaluate the impact of the COVID-19 pandemic and stewardship implementation, assess diagnostic performance, describe antifungal treatment patterns, and examine clinical outcomes over a ten-year period.

## 2. Materials and Methods

### 2.1. Study Design and Setting

This retrospective observational study included all episodes of candidemia (*n* = 306) diagnosed at Hospital Universitario Central de Asturias (HUCA), a 1.000-bed tertiary-care hospital in Oviedo, Spain, serving a catchment population of approximately 1 million inhabitants. The study was conducted over a 10-year period (2015–2024). HUCA provides comprehensive medical and surgical care, including specialized intensive care units and hematology-oncology services.

### 2.2. Case Definition and Study Periods

Candidemia was defined as the isolation of *Candida* spp. or phylogenetically related yeasts from at least one blood culture in a patient with compatible clinical findings. Episodes were stratified into two periods (2015–2019 and 2020–2024) to evaluate temporal trends. Polymicrobial candidemia was defined as the recovery of ≥2 yeast species from the same episode.

Recurrent candidemia was defined as a new episode occurring after documented microbiological clearance and clinical resolution of the index episode. Early recurrence caused by the same species and associated with a related presumed source was considered relapse, whereas episodes caused by a different species and/or associated with a different presumed source were classified as reinfection. These definitions were adapted from previously published studies on recurrent candidemia [23].

### 2.3. Variables and Data Sources

Microbiological data were obtained from the laboratory information system (Clinisys™ GestLab, Woking, UK) by selecting all blood cultures positive for yeasts during the study period. Clinical data were collected through review of electronic medical records (Cerner Millennium^®^, Kansas City, MO, USA).

All study variables were recorded in a standardized case report form (CRF). Demographic variables included age and sex. Clinical variables comprised the hospital service responsible for patient management, underlying comorbidities, immunosuppression status, predisposing factors for candidemia, the presumed source of infection, previous exposure to antibacterial agents, antifungal prophylaxis, and the presence of concomitant infections during the candidemia episode.

Microbiological variables included the date of blood culture collection, the causative yeast species, antifungal susceptibility results, blood culture time-to-positivity, time to species identification by conventional culture and MALDI-TOF MS, and, when available, time to identification by multiplex PCR. Therapeutic variables included empirical and targeted antifungal treatment, treatment duration, antifungal modifications during follow-up, and Antimicrobial Stewardship Program (ASP) interventions. Outcome variables included crude all-cause in-hospital mortality during the index hospitalization. Because of the retrospective design of the study, candidemia-attributable mortality was not assessed.

### 2.4. Microbiological Methods

Throughout the study period, blood cultures were processed using an automated incubation and reading system. Each set consisted of one aerobic and one anaerobic bottle. In pediatric patients for whom the standard sample volume (10 mL) could not be obtained, a specific pediatric bottle was used.

From 2015 to 2018, the BD BACTEC™ FX system (Becton Dickinson, Franklin Lakes, NJ, USA), which detects bacterial and fungal growth by fluorescence, was used. From 2019 to 2024, the BACT/ALERT^®^ system (bioMérieux, Marcy-l’Étoile, France), which detects growth using colorimetric technology, was employed.

Positive blood cultures were subcultured onto Sabouraud agar and CHROMagar™Candida (bioMérieux) and incubated aerobically at 30 °C until growth. Species-level identification was performed by matrix-assisted laser desorption/ionization time-of-flight mass spectrometry (MALDI-TOF, Bruker Daltonics). Only during the first year (2015), biochemical testing with API 32 C^®^ (bioMérieux) was employed in cases where MALDI-TOF identification was challenging.

Antifungal susceptibility testing was performed in all isolates using the commercially available broth microdilution system Sensititre YeastOne^®^ (Thermo Fisher Scientific, Cleveland, OH, USA). Minimum inhibitory concentrations (MICs) were interpreted according to the Clinical and Laboratory Standards Institute (CLSI) reference methodology for broth microdilution antifungal susceptibility testing of yeasts (document M27) [24,25] and the most recent CLSI species-specific clinical breakpoints and epidemiological cutoff values (ECVs) published in CLSI document M60 [26], which were routinely applied for susceptibility categorization in our laboratory.

PCR-based identification was performed using the BioFire^®^ Blood Culture Identification 2 panel (BCID2; BioFire Diagnostics/BioMérieux, Marcy-l’Étoile, France), a multiplex PCR assay designed for the rapid identification of bacterial and fungal pathogens directly from positive blood cultures. The fungal targets relevant to the present study included *Candida albicans*, *Nakaseomyces glabrata*, *Pichia kudriavzevii*, *Candida parapsilosis*, *Candida tropicalis*, *Candidozyma auris*, and *Cryptococcus neoformans/gattii*. The assay was introduced in our laboratory in 2018 and was initially applied in selected cases. From 2023 onward, BCID2 was incorporated into routine practice and systematically performed whenever yeast was observed on Gram stain following blood culture positivity. Overall, PCR-based identification was performed in 84 candidemia episodes during the study period.

### 2.5. Antimicrobial Stewardship Program (ASP)

A multidisciplinary Antimicrobial Stewardship Program (ASP) was established at HUCA in 2019 and operated through a prospective audit-and-feedback model. Following microbiological confirmation of candidemia, each case underwent structured review by a multidisciplinary team comprising infectious diseases and internal medicine physicians, clinical microbiologists, intensive care specialists, hospital pharmacists, pediatricians, emergency physicians, and members of the primary treating service. The ASP provided individualized recommendations regarding empirical and targeted antifungal therapy, escalation or de-escalation strategies, treatment duration, follow-up blood cultures, source-control interventions, including central venous catheter removal whenever feasible, and assessment for metastatic complications such as ocular or cardiac involvement. Recommendations were communicated directly to the treating physicians and documented in the electronic medical record, facilitating timely translation of microbiological findings into evidence-based clinical decision-making.

Importantly, the ASP was aligned with the principles outlined in the recent ECMM/ISHAM/ASM Global Guideline for the Diagnosis and Management of Candidiasis, which emphasizes the integration of rapid diagnostics, diagnostic stewardship, antifungal stewardship, source control, and multidisciplinary management as key components of optimal candidemia care [20]. Consistent with these recommendations, stewardship interventions in our institution focused not only on optimizing antifungal selection, escalation, and de-escalation strategies, but also on ensuring adherence to evidence-based management practices, including follow-up blood cultures to document microbiological clearance, timely catheter removal, screening for metastatic complications, and appropriate treatment duration. By systematically reinforcing these measures and integrating microbiological data into clinical decision-making, the ASP contributed to standardizing candidemia management across specialties and promoting guideline-concordant care throughout the study period [20].

### 2.6. Statistical Analysis

Statistical analyses were performed to assess the significance of observed differences. IBM SPSS Statistics version 21.0 and Microsoft Excel 2010 were used for statistical analyses and figure generation. Comparisons between periods (2015–2019 vs. 2020–2024) were performed using Fisher’s exact test for categorical variables. A two-sided *p*-value <0.05 was considered statistically significant for all hypothesis tests. Results should be interpreted as descriptive and hypothesis-generating.

### 2.7. Ethics

The collected data were entered into the CRF after patient anonymization. Each patient was dissociated from identifiable information and linked to a code known only to the investigator. This process ensured compliance with data protection regulations (Organic Law 3/2018, of December 5, on the Protection of Personal Data and guarantee of digital rights). Given the retrospective nature of the study, the anonymization of data, and the impracticability of obtaining consent from all patients, a waiver of informed consent was requested from the Research Ethics Committee of the Principality of Asturias (CEImPA 2025.323).

## 3. Results

### 3.1. Incidence and Temporal Trends

**Figure 1:**
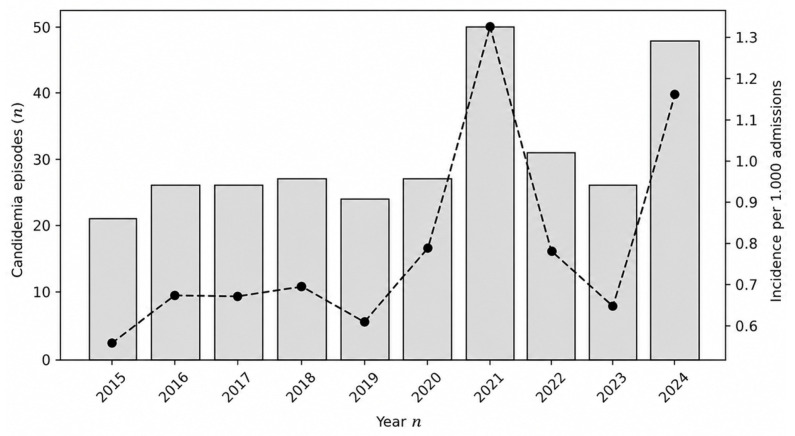
Annual number of candidemia episodes (bars) and incidence per 1.000 hospital admissions (dashed line) at Hospital Universitario Central de Asturias (HUCA), Oviedo, Spain, during the 10-year study period (2015–2024; *n* = 306 episodes).

A total of 306 candidemia episodes were identified at the Hospital Universitario Central de Asturias over a ten-year study period (2015–2024). The mean incidence was 0.79 episodes per 1.000 admissions. Annual incidence remained relatively stable (21–27 episodes/year) over the first half of the study period, until 2020, followed by a marked increase during and after the COVID-19 pandemic with a first peak in 2021 (*n* = 50) and a second peak in 2024 (*n* = 48). Figure 1 summarizes annual episode counts and incidence per 1.000 admissions.

Of the total episodes, 297 (97.1%) were caused by *Candida* spp. or closely related yeasts, while 9 episodes involved other yeasts (*Cryptococcus*, *Rhodotorula*, *Wickerhamomyces*, and *Pichia*), reflecting the broader spectrum of fungemia in hospitalized patients.

These episodes occurred in 305 patients; only one patient experienced more than one candidemia episode. This was a 62-year-old woman admitted for lumbar arthrodesis, who developed an initial episode due to *Candida albicans* during her stay in the intensive care unit (ICU), with the central venous catheter (CVC) in the jugular vein identified as the most probable source. Following antifungal therapy, CVC removal, and resolution, she experienced a second episode due to *Candida parapsilosis* during admission to the Traumatology service (CVC in the subclavian vein), notable for fluconazole resistance with a minimum inhibitory concentration (MIC) of 8 µg/mL.

### 3.2. Etiology and Species Distribution

To characterize the etiological agents, episodes were analyzed for the entire study period (2015–2024) and then stratified into two periods (2015–2019 vs. 2020–2024) in order to analyze temporal trends.

Overall, 292/306 (95.4%) of episodes were monofungal (caused by a single yeast species), whereas 14/306 (4.6%) had a polyfungal etiology.

**Figure 2:**
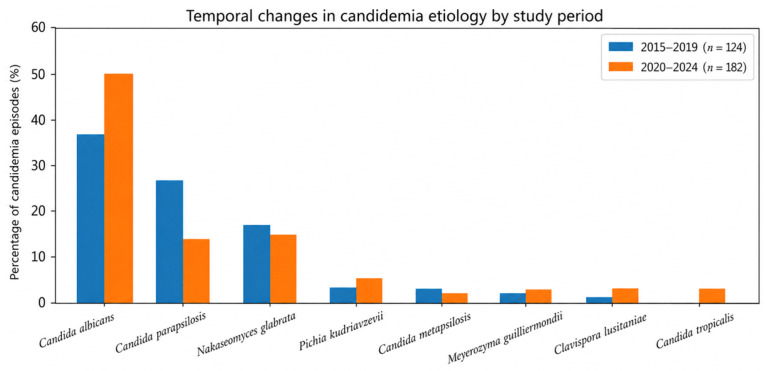
Changes in the distribution of the most common yeast species causing candidemia at Hospital Universitario Central de Asturias (HUCA), Oviedo, Spain, during the 10-year study period, comparing 2015–2019 (*n* = 124 episodes) and 2020–2024 (*n* = 182 episodes).

The distribution of yeast species causing monofungal and polyfungal candidemia across the entire study period and by five-year intervals is showing in Table 1 and Figure 2.

*C. albicans* was the most frequently isolated species (44.8%) followed by *C. parapsilosis* sensu stricto (19.0%), *N. glabrata* (15.7%) and *P. kudriavzevii* (4.2%). Among non-*Candida* genera, *Cryptococcus neoformans* was notable (*n* = 5). There were two episodes due to *Rhodotorula mucilaginosa*, and one each due to *Wickerhamomyces anomalus* and *Pichia cactophila*.

As illustrated in Table 1 and Figure 2 a significant shift in species distribution was observed between periods: *C. albicans* increased significantly from 37.1% to 50% of isolates (*p* = 0.027). *C. parapsilosis* sensu stricto, the second-most frequent species in 2015–2019, became third in 2020–2024 due to a significant decline (26.6% vs. 13.7%, *p* = 0.007).

Notably, *C. parapsilosis* was the leading species in pediatric patients (<18 years), accounting for 5 of 7 isolates. Relatively increase in *N. glabrata* in the second period, becoming the second most common species (*p* = 0.05). Isolates of *P. kudriavzevii* also rose significantly (*p* < 0.05). Other *Candida* species increased significantly from 11 isolates (8.3%) in the first period to 25 (13.2%) in the second, particularly *C. tropicalis* and *Clavispora lusitaniae* (*p* < 0.05). This shift is clinically relevant given differences in antifungal susceptibility profiles and therapeutic implications.

Most *C. neoformans* episodes (4 of 5) occurred in 2015–2019, as did both *R. mucilaginosa* infections. With one exception, all occurred in immunosuppressed patients. Although three episodes involved onco-hematologic patients, the degree of immunosuppression indicates that any clinical service may be affected by these lesser common infections. Among *C. neoformans* cases, only two occurred in patients with HIV/AIDS; two were in kidney transplant recipients, and one in a patient with cirrhosis.

Of the polyfungal episodes (4.6%) involving more than one yeast species, summarized in Table 1, with *C. albicans* + *C. parapsilosis* (*n* = 4) being the most frequent combination. Polyfungal candidemia remained uncommon and stable across periods, with seven episodes occurred in 2015–2019 and seven in 2020–2024.

Candidemia with concurrent bacteremia in the same blood culture accounted for 30.5% of episodes, consistent with prior reports [27].

### 3.3. Clinical Setting: Distribution of Candidemia Episodes by Clinical Service

To better understand the clinical setting in which candidemia was diagnosed, episodes were categorized according to the type of hospital service responsible for patient management. Overall, candidemia occurred most frequently in medical wards (117/306 episodes, 38.2%), followed by intensive care units (97/306, 31.7%) and surgical wards (92/306, 30.1%). Thus, candidemia was distributed relatively evenly across the three major hospital settings, with a slight predominance in medical wards.

A significant increase in ICU-associated candidemia was observed in 2020–2024, from 27/124 (21.8%) in 2015–2019 to 70/182 (38.5%) in 2020–2024 (*p* = 0.003), coinciding with the COVID-19 period.

**Figure 3:**
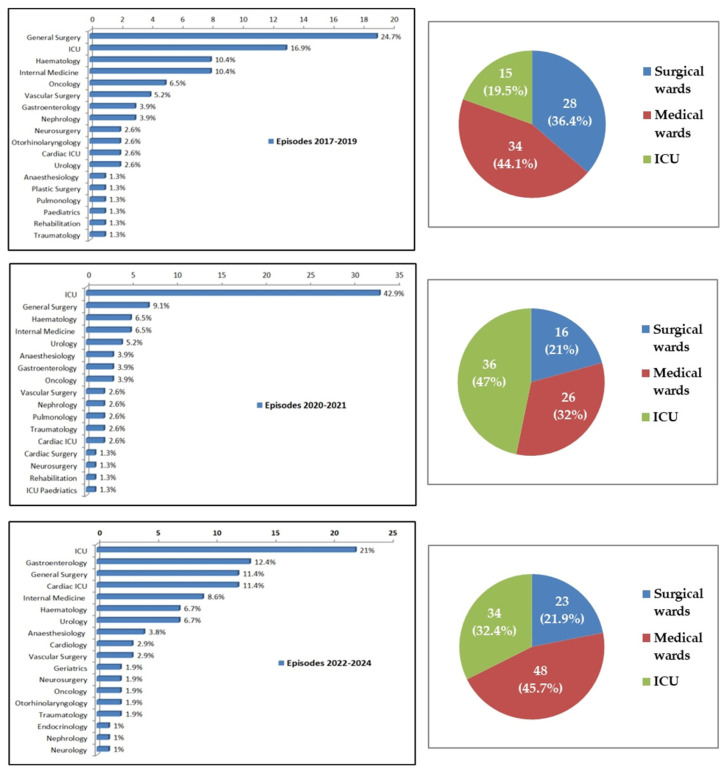
Distribution of candidemia episodes according to the responsible hospital service (left panels) and the major clinical setting (medical wards, surgical wards, and intensive care units; right panels) at Hospital Universitario Central de Asturias (HUCA), Oviedo, Spain, comparing the pre-pandemic (2017–2019), pandemic (2020–2021) and post-pandemic (2022–2024) periods. Footnote: The left panels show the percentage distribution of candidemia episodes among individual hospital services, whereas the right panels summarize episodes according to the three major clinical settings: medical wards, surgical wards, and intensive care units (ICUs).

To assess the impact of the COVID-19 pandemic, the study period was divided into three equal intervals (2017–2019, 2020–2021, and 2022–2024), corresponding to the three years preceding the pandemic, the pandemic years, and the three post-pandemic years (Figure 3).

Figure 3 clearly shows a significant increase in ICU-diagnosed episodes during the pandemic years (2020–2021) (*p* < 0.05), accompanied by a decrease across other services. Particularly noteworthy is the decline in General Surgery, from 24.7% in 2017–2019 to 9.1% during the pandemic (*p* < 0.05). In the post-pandemic period (2022–2024), the distribution returned to a pattern similar to the pre-pandemic years, with increases in services such as Urology and Gastroenterology (*p* < 0.05), which are discussed later.

In summary, the departmental distribution of candidemia evolved considerably across the three phases of the COVID-19 era, with distinct epidemiological patterns observed during the pre-pandemic (2017–2019), pandemic (2020–2021), and post-pandemic (2022–2024) periods. Stratified analyses showed an increased ICU burden during pandemic years and a redistribution toward surgical and medical services post-pandemic. The significant increase in ICU-associated candidemia highlights the strong relationship between critical illness and invasive fungal infections. This finding likely reflects increased device use, immunosuppression, and antibiotic exposure during the pandemic.

### 3.4. Patient Profile and Predisposing Factors

The following host factors were analyzed: age, sex, comorbidities, immune status, candidemia risk factors, and most likely infectious focus.

**Figure 4:**
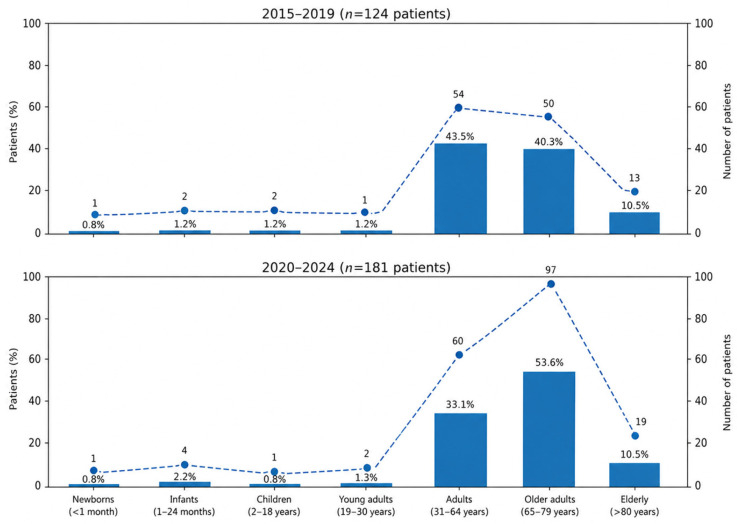
Age distribution of patients with candidemia at Hospital Universitario Central de Asturias (HUCA), Oviedo, Spain, comparing the 2015–2019 (*n* = 124 patients) and 2020–2024 (*n* = 181 patients) study periods. Bars represent the percentage of patients within each age group, whereas the dashed line indicates the corresponding number of patients. Age analyses were performed at the patient level (*n* = 305), as one patient experienced two distinct candidemia episodes during the study period.

Most patients were aged ≥65 years, representing 179 of 305 patients (58.7%) (Figure 4). The proportion of patients aged ≥65 years increased significantly from 50.8% in 2015–2019 to 64.1% in 2020–2024 (*p* < 0.05). In contrast, pediatric patients (<18 years) accounted for only 7 of 305 patients (2.3%) in the overall cohort.

Mean age increased significantly from 60.6 to 66.1 years (*p* < 0.05). Demographic analyses were performed at the patient level because one individual experienced two distinct candidemia episodes during the study period. Overall, 215 of 305 patients (70.5%) were male and 90 of 305 (29.5%) were female (*p* < 0.05). Throughout the study period, the proportion of male patients consistently exceeded that of female patients, reaching a maximum of 85.2% in 2018 and a minimum of 57.1% in 2015.

Patients with candidemia frequently presented with multiple underlying comorbidities. Although hypertension was the most commonly reported condition in our cohort, it is not considered a specific risk factor for candidemia. In contrast, several well-recognized predisposing conditions were highly prevalent, including diabetes mellitus (27.9%), solid malignancy (27.9%), ischemic heart disease and/or valve replacement (14.4%), hematologic malignancy (11.5%), and obesity (10.8%). The most common comorbidities identified in our cohort are summarized in Table 2.

Overall, 22 of 305 patients (7.2%) had undergone transplantation, including kidney (*n* = 9), hematopoietic stem-cell (*n* = 7), and liver (*n* = 4) transplantation; one patient had undergone lung transplantation and one heart transplantation.

Regarding solid tumors, the most frequent were colon adenocarcinoma (*n* = 20), pancreatic adenocarcinoma (*n* = 9), and urothelial carcinoma of the bladder (*n* = 8). Among hematologic malignancies, the most frequent were acute myeloid leukemia (*n* = 12), myelodysplastic syndrome (*n* = 5), and follicular non-Hodgkin lymphoma (*n* = 3).

Overall, immunosuppression was present in 177 of 305 patients (58.0%) and increased significantly in the later period, from 58 of 124 patients (46.8%) in 2015–2019 to 119 of 181 patients (65.7%) in 2020–2024 (*p* = 0.001), particularly due to corticosteroid and immunosuppressive therapies (Table 3 and Table 4). Less frequent factors included severe malnutrition and prematurity. Notably, there was a significant increase in corticosteroid use (52.5% vs. 23.4% of all patients) and in other immunosuppressants (13.8% vs. 5.6%) in 2020–2024 compared with 2015–2019 (both *p* < 0.05). Use of monoclonal antibodies also rose (*n* = 13 vs. 2). Most HIV/AIDS cases occurred in 2015–2019 (six of eight patients).

Regarding candidemia risk factors, the most frequent was central venous catheter use (CVC; *n* = 223; 73.1% of all patients), followed by others included urinary catheterization (*n* = 77; 25.2%), abdominal surgery (*n* = 50; 16.4%), abdominal drainage (*n* = 45; 14.8%), mechanical ventilation (*n* = 26; 8.5%), and parenteral nutrition (*n* = 21; 6.9%) increased significantly in 2020–2024. Among central venous access, subclavian CVCs were most frequent (*n* = 82), followed by jugular CVCs (*n* = 68).

Meanwhile, central venous catheterization remained stable across periods (71.0% vs. 74.2%). In contrast, in 2020–2024, there was a significant increase relative to 2015–2019 in patients with urinary catheters (34.1% vs. 12.1%) and those requiring mechanical ventilation (13.2% vs. 1.6%) (both *p* < 0.05); while abdominal surgery and abdominal drainage decreased (Table 4).

Identifying the focus of candidemia is sometimes challenging. The most frequent source was the CVC (49.8% of episodes; *n* = 152), followed by abdominal focus (*n* = 41; 13.4%), urinary catheter (*n* = 28; 9.2%), combined abdominal/CVC (*n* = 18; 5.9%), peripheral catheter (*n* = 12; 3.9%), nephrostomy (*n* = 11; 3.6%), and respiratory focus (*n* = 10; 3.3%). Of note, urinary catheterization as the most likely source increased significantly in the second period (13.7% vs. 2.4%) (*p* < 0.05).

### 3.5. Diagnostic Turnaround Time

To assess diagnostic performance in candidemia, we analyzed the following variables: time to blood culture positivity (hours), time to species identification by culture and mass spectrometry (hours), and time to identification by PCR (hours).

**Figure 5:**
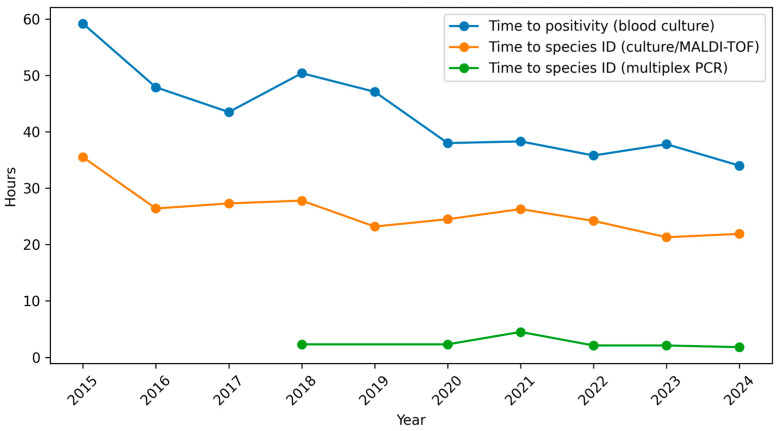
Changes in diagnostic turnaround times for candidemia at Hospital Universitario Central de Asturias (HUCA), Oviedo, Spain, from 2015 to 2024, including time to blood culture positivity, time to species identification by culture/MALDI-TOF MS, and time to species identification by multiplex PCR.

As shown in Figure 5, significant improvements in diagnostic performance were observed during the study period. The time to blood culture positivity, measured to the first positive bottle, decreased significantly between the two periods 49.6 h vs. 36.8 h in the later period (*p* < 0.05). The mean time-to-positivity was 41.7 h (range, 6.5–155 h; median, 36.2 h).

Only three episodes had time-to-positivity exceeding five days—*R. mucilaginosa* (128 h), *N. glabrata* (129 h), and *C. albicans* (155 h)—suggesting that the standard five-day incubation protocol used by most laboratories is adequate, as 99% of candidemia cases would have been detected within this timeframe.

The mean time to species identification by MALDI-TOF following growth on solid media was 25.4 h (range, 9–94 h; median, 22.9 h). Main times to species identification by MALDI-TOF also decreased significantly in the second period (23.6 h) than in the first (28 h) (*p* < 0.05).

PCR-based identification after blood culture positivity was performed using the *BioFire^®^ Blood Culture Identification 2* (BCID2) panel in 84 candidemia episodes. The assay was introduced in 2018 and initially reserved for selected cases; however, from 2023 onward it was implemented routinely for all yeast-positive blood cultures. Consequently, 66 of the 84 PCR-based identifications (78.6%) were performed during the 2023–2024 period. The mean time to PCR result was 2.3 h (range, 1.2–14 h; median, 1.7 h) (Figure 5).

Compared with culture, multiplex PCR showed a sensitivity of 91.7% at the episode level; this decreased to 87.4% when identification of all yeast species was required. The reduction reflects the presence of species not included in the multiplex panel (e.g., *Clavispora lusitaniae* *n* = 4, *Kluyveromyces marxianus* *n* = 2, *Candida orthopsilosis* *n* = 2, *Candida metapsilosis* *n* = 2, and *Meyerozyma guilliermondii* *n* = 1). PCR performance was limited by panel coverage, particularly in rare species.

A progressive reduction in diagnostic turnaround time was observed, including shorter time to blood culture positivity and faster species identification. The introduction of multiplex PCR enabled rapid identification within hours after positivity, representing a major improvement in diagnostic efficiency.

However, PCR performance was limited by panel coverage, particularly for uncommon species, highlighting the need for complementary diagnostic approaches.

### 3.6. Antifungal Susceptibility

Antifungal susceptibility testing was routinely performed for all yeast isolates recovered from positive blood cultures during the study period. Susceptibility profiles varied considerably according to species and antifungal class.

Fluconazole resistance was highest among *Nakaseomyces glabrata* isolates, affecting 25 of 52 isolates (48.1%). Lower resistance rates were observed in *Candida parapsilosis complex* (5/72; 6.9%) and *Candida albicans* (6/146; 4.1%). Fluconazole resistance was also detected in *Meyerozyma guilliermondii* (2/8; 25%) and *Cryptococcus neoformans* (3/5; 60%). As expected, all *Pichia kudriavzevii* (*Candida krusei*) isolates were resistant to fluconazole (15/15; 100%). No fluconazole resistance was observed among *Candida tropicalis* isolates.

Voriconazole resistance was identified predominantly among *N. glabrata* isolates (17/52; 32.7%), followed by *P. kudriavzevii* (5/15; 33.3%), *C. albicans* (5/146; 3.4%), and *M. guilliermondii* (1/8; 12.5%). All *Rhodotorula mucilaginosa* isolates exhibited resistance to voriconazole.

Reduced susceptibility to echinocandins was observed mainly among *Candida parapsilosis complex* isolates, with 17 of 72 isolates (23.6%) categorized as susceptible dose-dependent (SDD) to anidulafungin. Acquired echinocandin resistance was uncommon among Candida species and was identified only in a single *Meyerozyma guilliermondii* isolate (1/8; 12.5%). As expected, intrinsic echinocandin resistance was observed in *Cryptococcus neoformans* (5/5; 100%), *Rhodotorula mucilaginosa* (2/2; 100%), *Wickerhamomyces anomalus* (1/1; 100%), and *Pichia cactophila* (1/1; 100%).

Resistance to amphotericin B was rare among the most frequently isolated *Candida* species. However, all *Clavispora lusitaniae* isolates (7/7; 100%) were resistant, consistent with the known intrinsic or species-associated reduced susceptibility of this organism. Resistance was also observed in one *Kluyveromyces marxianus* isolate (1/3; 33.3%). Resistance to 5-fluorocytosine was detected in one *Cryptococcus neoformans* isolate (1/5; 20%).

### 3.7. Antifungal Therapy and Stewardship (ASP)

A total of 33 patients (10.8%) had received antifungal prophylaxis prior to the candidemia episode, and an additional 8 patients were already undergoing antifungal treatment. Among those receiving prophylaxis, 18 were hematologic patients (54.5%).

Eleven of the patients who had received prior prophylaxis or antifungal therapy were found to harbor species resistant to—or with elevated minimum inhibitory concentrations (MICs) against—the antifungal agent used: *Pichia kudriavzevii* (*n* = 4, fluconazole), *Nakaseomyces glabrata* (*n* = 3, fluconazole, voriconazole, isavuconazole), *Wickerhamomyces anomalus* (*n* = 1, posaconazole), *Rhodotorula mucilaginosa* (*n* = 1, posaconazole), *Candida parapsilosis* (*n* = 1, fluconazole), and *Pichia cactophila* (*n* = 1, fluconazole). These cases may be classified as breakthrough invasive fungal infections (IFI).

**Figure 6:**
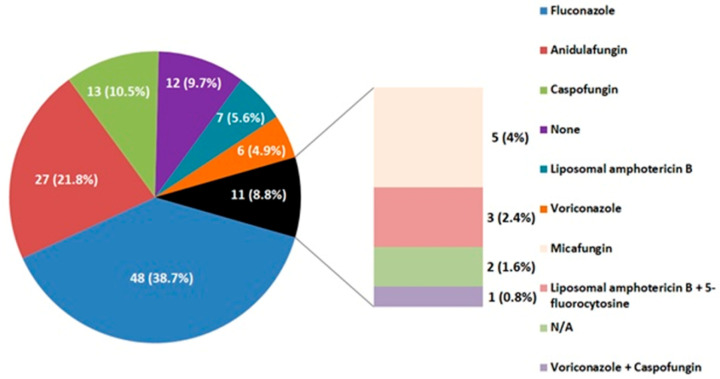

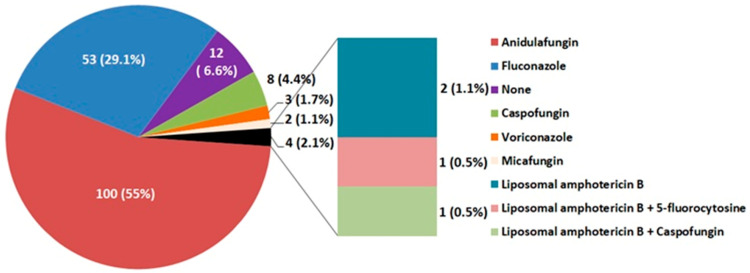
Changes in empirical antifungal prescribing patterns for candidemia at Hospital Universitario Central de Asturias (HUCA), Oviedo, Spain, comparing the 2015–2019 (*n* = 124 episodes) (**top**) and 2020–2024 (*n* = 182 episodes) (**bottom**) study periods.

Among the 306 candidemia episodes included in the study, empirical antifungal treatment was administered in 280 episodes (91.5%). In 24 episodes (7.8%), candidemia was diagnosed postmortem and no empirical antifungal therapy was administered, while two episodes (0.7%) occurred in patients who had been transferred to another hospital before the microbiological diagnosis was established and therefore did not receive antifungal treatment at our center (Figure 6).

Empirical antifungal treatment strategies changed substantially over the study period. Overall echinocandin use increased significantly from 36.3% in 2015–2019 to 60.5% in 2020–2024 (*p* < 0.05). This trend was largely driven by a marked increase in anidulafungin use, from 27 cases (21.8%) in the first period to 100 cases (55.0%) in the second period (*p* < 0.05). These findings reflect a progressive shift toward echinocandin-based empirical therapy in more recent years, consistent with evolving guideline recommendations and changes in the epidemiology of candidemia. 

Targeted therapy patterns also changed: targeted fluconazole use increased significantly from 38/124 (30.6%) in the first period to 81/124 (44.5%) in the second (*p* < 0.05). Anidulafungin use also rose from 29/124 (23.4%) to 51/124 (28%) (*p* = 0.05).

The mean duration of antifungal therapy was 18.5 days (range, 1–167 days; median, 15.5 days). Almost all patients (98.4%; *n* = 301) had received prior broad-spectrum antibiotics before the onset of candidemia.

**Figure 7:**
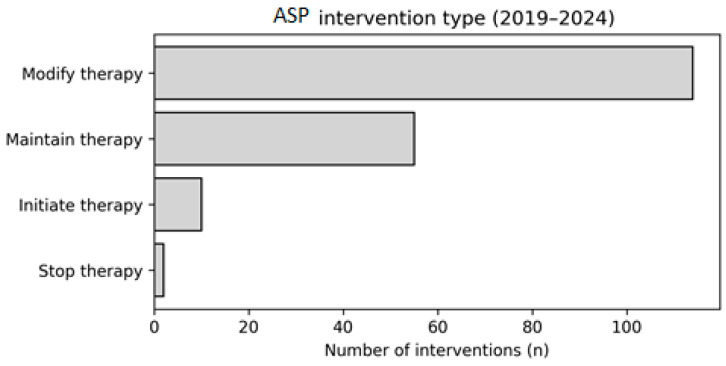
Types of Antimicrobial Stewardship Program (ASP) interventions performed in candidemia episodes at Hospital Universitario Central de Asturias (HUCA), Oviedo, Spain, during 2019–2024 (*n* = 181 reviewed episodes).

The Antimicrobial Stewardship Program (ASP), implemented at our hospital in 2019, conducted 181 interventions in candidemia cases. The most frequent intervention was recommendation of antifungal modification, followed by maintaining the empirical treatment when deemed appropriate (Figure 7).

The standard recommendation for each candidemia episode includes maintaining antifungal treatment for 14 days after the first negative blood culture, performing a follow-up blood culture 48–72 h after starting therapy, removing the central venous catheter (CVC), and conducting both a transthoracic echocardiogram (TTE) and a fundoscopic eye examination.

**Figure 8:**
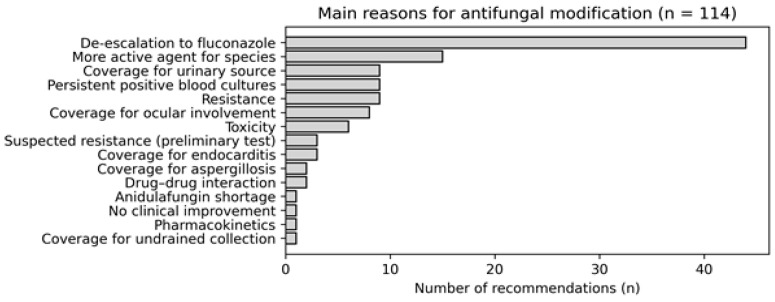
Main reasons for antifungal treatment modification following Antimicrobial Stewardship Program (ASP) review of candidemia episodes at Hospital Universitario Central de Asturias (HUCA), Oviedo, Spain, during 2019–2024 (*n* = 114 recommendations).

Between 2019 and 2024, ASP group reviewed 181 candidemia cases and issued recommendations. Most interventions recommended modifying the antifungal regimen (63.0%), followed by maintaining therapy (30.4%). The most common reason for modification was de-escalation to fluconazole after stabilization and microbiological confirmation of susceptibility (Figure 8). These findings indicate a shift toward guideline-consistent antifungal prescribing.

### 3.8. Complications and Outcomes

During the study period, metastatic complications included five cases of endocarditis (1.6%) were diagnosed—four based on clinical and imaging criteria and one confirmed by isolation of *C. parapsilosis* from an aortic valve. Additionally, 12 episodes (3.9%) presented ocular involvement: five cases of retinitis, three of endophthalmitis, two with unspecified ocular involvement, one subretinal abscess, and one corneal ulcer. One case of epidural abscess due to *C. parapsilosis* was also reported. These conditions required prolonged antifungal treatment, strict source control, and close follow-up to prevent secondary complications—management strategies coordinated by the ASP group.

**Figure 9:**
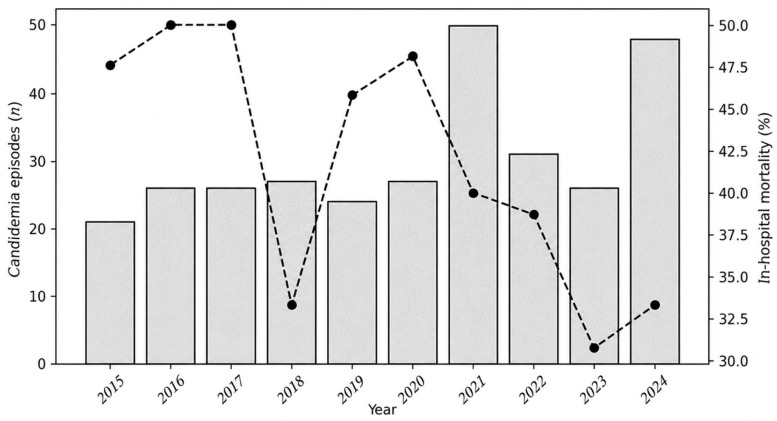
Annual number of candidemia episodes (bars) and crude in-hospital mortality (dashed line) at Hospital Universitario Central de Asturias (HUCA), Oviedo, Spain, during the 10-year study period (2015–2024; *n* = 306 episodes).

Crude in-hospital mortality was analyzed among patients who experienced candidemia. Overall, 125 of 306 episodes (40.8%) were associated with in-hospital death during the index hospitalization. Mortality rates varied over time, as shown in Figure 9. The highest crude in-hospital mortality rates were observed in 2016 and 2017 (50.0%), followed by fluctuations throughout the study period, including a peak of 48.1% in 2020. During the final two years of the study, crude in-hospital mortality declined significantly, reaching 30.8% in 2023 and 33.3% in 2024 (*p* < 0.05). This reduction temporally coincided with improvements in diagnostic turnaround times and greater stewardship activity. However, given the observational nature of the study, no causal relationship can be established.

## 4. Discussion

This study provides a comprehensive longitudinal assessment of candidemia, capturing dynamic changes in epidemiology, diagnostic workflows acceleration, antifungal susceptibility, prescribing patterns and outcomes in the context of the COVID-19 period and the implementation of an institutional Antimicrobial Stewardship Program (ASP). Beyond documenting shifts in species distribution and clinical setting, our findings highlight keys modifiable determinants of candidemia care: (i) clinically actionable integration of rapid diagnostics and (ii) antifungal stewardship within a structured clinical management pathway that translates microbiology results into timely therapeutic and source-control decisions. The findings highlight the dynamic nature of candidemia and the importance of modifiable factors in its management.

The average incidence of candidemia during the ten-year period was 0.79 episodes per 1.000 admissions, comparable to other studies in Spain, such as the CANDIPOP study (0.89/1.000 admissions) [28], and lower than reports from Latin America (1.18/1.000 admissions) [29].

Two prominent peaks were observed in 2021 (*n* = 50) and 2024 (*n* = 48). The increase in candidemia episodes observed after 2020 may reflect external healthcare pressures associated with the pandemic and post-pandemic periods, including changes in patient case-mix, ICU occupancy, use of invasive devices, and healthcare delivery, rather than a purely intrinsic epidemiological cycle. Nevertheless, this interpretation should be considered cautiously given the retrospective single-center design of the study.

The first coincided with the COVID-19 pandemic, approximately one year after its onset. The pandemic has been reported to cause a significant rise in invasive fungal infections among hospitalized patients, particularly candidiasis and aspergillosis in Europe [11]. The 2024 peak is more difficult to interpret. That year recorded the highest number of hospital admissions (*n* = 41,271), 2.650 more than the ten-year average (mean: 38,621). Comparing 2023 and 2024, an increase in candidemia episodes was noted across several months—June and July (5 in 2023 vs. 12 in 2024), as well as September, October, December, and March—indicating that the rise was not confined to a specific season. No single medical service showed a statistically significant increase compared with the previous year. Possible explanations include staff turnover during that period. Interestingly, data from 2025 (up to September 9) recorded 19 episodes; extrapolating to year-end would yield figures consistent with previous years, suggesting that the 2024 increase was an isolated event.

The observed shift in species distribution—characterized by an increased proportion of *C. albicans* and a decline in *C. parapsilosis*, alongside the consolidation of *N. glabrata* as the second most frequent pathogen—has direct therapeutic implications. While *C. albicans* remains broadly susceptible to azoles, the increasing clinical relevance of *N. glabrata* has important therapeutic implications due to its reduced susceptibility and frequent resistance to azole antifungals, particularly fluconazole. Therefore, current international guidelines recommend echinocandins as the preferred initial therapy for candidemia, especially in critically ill or immunocompromised patients. Subsequent step-down to azole therapy should be guided by species identification and antifungal susceptibility testing to ensure adequate antifungal coverage [18,30].

The predominance of *C. albicans* in our cohort may be related to epidemiological changes observed during the COVID-19 pandemic. In a large multicenter U.S. study of COVID-19-associated candidemia, *C. albicans* was the most frequently isolated species, accounting for approximately 44% of cases, while the overall incidence of candidemia increased among critically ill patients. [12,31]. This contrasts with other findings, such as those of Tanriverdi and colleagues [10], who reported an increase in *C. parapsilosis* in a seven-year Turkish study—underscoring the geographic variability in candidemia etiology.

These findings demonstrated that shifts toward non-albicans *Candida* species are closely linked to antifungal exposure, ICU burden, and evolving healthcare practices, with resistance trends increasingly shaping empirical therapy decisions. Such studies, like our study, emphasize that local epidemiology must continuously inform antifungal stewardship policies rather than relying solely on historical patterns.

During the pandemic years (2020–2021), a substantial increase in ICU-associated candidemia episodes was observed (42.9%), accompanied by a decline in cases diagnosed in other hospital departments. This pattern may be attributable to the marked reduction in elective procedures and non-urgent hospital activity during the peak phases of the COVID-19 pandemic. In contrast, the post-pandemic period (2022–2024) was characterized by a marked decline in ICU-associated candidemia cases (21.0%) and a concomitant redistribution of cases toward other clinical services. The largest increases were observed in the Gastroenterology (12.4% vs. 3.9%) and Cardiac Surgery (11.4% vs. 2.6%) departments. This shift likely reflects the progressive restoration of routine healthcare activity, including the resumption of elective surgical programs and specialized inpatient care, resulting in a return of candidemia cases to patient populations with distinct underlying risk profiles. Overall, the COVID-19 pandemic appears to have acted as an epidemiological accelerator, temporarily concentrating candidemia cases in critically ill ICU patients and increasing exposure to device-related risk factors before a subsequent redistribution across other hospital departments as routine clinical activity resumed.

Older age (≥65 years) was a strong risk factor, observed in 58.7% of cases, consistent with other studies [31]. Notably, pediatric cases (<18 years) represented only 2.3% of episodes (*n* = 5), a lower proportion than reported elsewhere—where the highest incidence per 100,000 population occurs in infants under one year [28]—highlighting effective management within our pediatric units. The increasing proportion of immunosuppressed patients, particularly those receiving corticosteroids or monoclonal antibodies, represents an elevated risk group for candidemia.

### 4.1. Rapid Diagnostics and Diagnostic Stewardship: Linking Turnaround Time to Outcomes

A central finding of this study is the progressive improvement in diagnostic turnaround time, included reduced time to blood culture positivity and faster species identification via MALDI-TOF, further enhanced by the implementation of multiplex PCR in recent years. This improvement may facilitate earlier and more targeted optimization of antifungal therapy, potentially improving clinical management.

From a clinical perspective, candidemia is a time-dependent infection where delays in initiating effective antifungal therapy and achieving source control are consistently associated with worse outcomes [3,4,19]. In our cohort, laboratory performance improved over time, with shorter blood culture time-to-positivity and faster species identification by culture/MALDI-TOF. Importantly, the introduction of multiplex PCR on yeast-positive blood cultures (implemented mainly in 2023–2024) achieved high episode-level sensitivity (91.7%) and enabled near real-time differentiation of clinically relevant species, including those with intrinsic or acquired resistance. However, limitations remain due to panel coverage, particularly for uncommon or emerging species.

Regarding diagnostics, the reduction in blood culture positivity time may partly reflect the change in automated culture systems but is unlikely to be the sole explanation. Neither system is specifically optimized for fungal detection, nor both rely on general bacterial and yeast media. Moreover, the systems were not used simultaneously, indicating that a parallel validation study would be useful.

The improvement in MALDI-TOF identification times observed in 2016 (from a mean of 36.5 h in 2015 to 26.4 h in 2016) corresponds with database enhancements implemented that year. The addition of more fungal spectral profiles improved identification accuracy and eliminated the need for backup biochemical tests. Sample preparation was also simplified by introducing formic acid for fungal cell wall disruption, expediting the process.

Routine implementation of PCR following positive Gram stains for yeasts (since 2023) further shortened identification times. Even negative PCR results provide valuable clinical guidance, suggesting the possibility of rare yeast species not included in the multiplex panel.

Importantly, the clinical value of rapid diagnostics lies not only in speed but in their integration into diagnostic stewardship framework, ensuring that results are translated into actionable clinical decisions. It is important to emphasize that diagnostic tools must be evaluated not in isolation but as components of a broader care pathway.

### 4.2. Antifungal Stewardship as an Implementation Driver

The implementation of a multidisciplinary Antimicrobial Stewardship Program (ASP) in 2019 represents a pivotal structural change in our institution. ASP provided structured recommendations and was associated with frequent antifungal optimization and reinforcement of key elements of candidemia care (follow-up cultures, early catheter removal, and appropriate treatment duration). Over the study period, antifungal prescribing patterns evolved significantly, with increased empirical use of echinocandins and more frequent targeted de-escalation to fluconazole following susceptibility confirmation.

This aligns with current international guidelines and reflects a shift toward precision antifungal therapy, recommending echinocandins as first-line therapy [17,19], balancing early effective treatment with minimization of unnecessary exposure.

ASP’s standard recommendations—such as obtaining control blood cultures 48–72 h after initiating therapy, treating for 14 days after the first negative culture, removing the CVC, and performing transthoracic echocardiography and fundoscopic examination—have improved candidemia management and clinician adherence in the hospital.

Although some authors argue that routine ophthalmologic examinations should be reserved for selected cases (e.g., ocular symptoms, persistent candidemia, severe immunosuppression, or non-verbal patients) [19], the detection of ocular involvement in 12 cases (3.9%) in our cohort supports the continuation of this low-cost preventive measure to avoid vision-threatening complications.

Rapid identification is only clinically impactful if it triggers action. In this sense, diagnostic stewardship (appropriate use of tests, rapid reporting, and direct communication of critical results) and antifungal stewardship are complementary. Although our study design does not allow causal inference, the decline in mortality observed in 2023–2024 (to approximately one-third of episodes) temporally paralleled the fastest diagnostic turnaround and the most established stewardship activity, supporting continued investment in rapid diagnostics integrated into clinical decision pathways.

More broadly, stewardship programs can reinforce a candidemia bundle approach (source control, follow-up cultures, and screening for complications), which is likely required to influence mortality beyond antifungal selection alone [17,19].

Finally, studies like this are valuable only if conducted periodically, as species distribution changes over time and across regions. Understanding which species predominate, in which departments, and among which patient populations is crucial for developing effective management protocols, preventing outbreaks, and reducing incidence. Dissemination of results across hospital departments allows implementation of corrective measures. Future work should include a prospective study and a multivariate analysis to identify risk factors independently associated with mortality.

### 4.3. Strengths and Limitations

The strengths of this study include its long ten-year observation period, the integration of microbiological data (including turnaround times and susceptibility profiles) with clinical data, and evaluation of real-world diagnostic and stewardship activity.

However, several limitations must be acknowledged. The retrospective single-center design limits generalizability, and the absence of severity-of-illness scores and time-to-effective therapy precludes risk-adjusted analyses. In addition, mortality was assessed as crude all-cause in-hospital mortality. Because of the retrospective design and the absence of predefined adjudication criteria, candidemia-attributable mortality could not be reliably determined. The observed reduction in mortality is encouraging but must be interpreted cautiously. While it temporally coincided with improvements in diagnostics and stewardship, causality cannot be established. Multiple confounding factors, including changes in patient population and healthcare practices, may have contributed.

## 5. Conclusions

Candidemia epidemiology and management evolved significantly over the study period, with notable changes in species distribution, diagnostic workflows, and antifungal prescribing patterns. The increasing burden in ICU settings and the emergence of resistant species highlight ongoing clinical challenges.

Improvements in diagnostic turnaround time and the implementation of antifungal stewardship strategies temporally coincided with reduced mortality, supporting the potential value of integrated diagnostic–stewardship approaches. However, their independent impact on clinical outcomes requires confirmation in future risk-adjusted studies.

Overall, this study supports the concept that optimal candidemia management requires an integrated approach combining rapid diagnostics, antifungal stewardship, and structured clinical pathways.

## Figures and Tables

**Table 1:** Distribution of yeast species causing candidemia episodes at Hospital Universitario Central de Asturias (HUCA), Oviedo, Spain, during the study period (2015–2024; *n* = 306 episodes), stratified by the 2015–2019 and 2020–2024 study periods.

Species	2015–2019 (*n* = 124)	2020–2024 (*n* = 182)	Total (*n* = 306)
*Candida albicans*	46 (37.1%)	91 (50.0%)	137 (44.8%)
*Candida parapsilosis*	33 (26.6%)	25 (13.7%)	58 (19.0%)
*Nakaseomyces glabrata*	21 (16.9%)	27 (14.8%)	48 (15.7%)
*Pichia kudriavzevii*	4 (3.2%)	9 (4.9%)	13 (4.2%)
*Candida metapsilosis*	3 (2.4%)	3 (1.6%)	6 (2.0%)
*Meyerozyma guilliermondii*	2 (1.6%)	4 (2.2%)	6 (2.0%)
*Clavispora lusitaniae*	1 (0.8%)	5 (2.7%)	6 (2.0%)
*Candida tropicalis*	0 (0.0%)	5 (2.7%)	5 (1.6%)
*Cryptococcus neoformans*	4 (3.2%)	1 (0.5%)	5 (1.6%)
*Rhodotorula mucilaginosa*	2 (1.6%)	0 (0.0%)	2 (0.7%)
*Kluyveromyces marxianus*	0 (0.0%)	2 (1.1%)	2 (0.7%)
*Candida orthopsilosis*	0 (0.0%)	2 (1.1%)	2 (0.7%)
*Wickerhamomyces anomalus*	1 (0.8%)	0 (0.0%)	1 (0.3%)
*Pichia cactophila*	0 (0.0%)	1 (0.5%)	1 (0.3%)
*Candida albicans + Candida parapsilosis*	3 (2.4%)	1 (0.5%)	4 (1.3%)
*Candida albicans + Nakaseomyces glabrata*	0 (0.0%)	2 (1.1%)	2 (0.7%)
*Nakaseomyces glabrata + Candida tropicalis*	2 (1.6%)	0 (0.0%)	2 (0.7%)
*Pichia kudriavzevii + Meyerozyma guilliermondii*	1 (0.8%)	0 (0.0%)	1 (0.3%)
*Candida albicans + Meyerozyma guilliermondii + Candida tropicalis*	1 (0.8%)	0 (0.0%)	1 (0.3%)
*Candida albicans + Candida tropicalis*	0 (0.0%)	1 (0.5%)	1 (0.3%)
*Candida albicans + Clavispora lusitaniae*	0 (0.0%)	1 (0.5%)	1 (0.3%)
*Candida orthopsilosis + Candida parapsilosis*	0 (0.0%)	1 (0.5%)	1 (0.3%)
*Pichia kudriavzevii + Kluyveromyces marxianus*	0 (0.0%)	1 (0.5%)	1 (0.3%)

Footnote: Values are presented as the number of episodes and percentage of all candidemia episodes within each study period. Polyfungal episodes are listed separately according to the species combination identified.

**Table 2:** Distribution of the most frequent comorbidities among patients with candidemia at Hospital Universitario Central de Asturias (HUCA), Oviedo, Spain, during the study period 2015–2024 (*n* = 305 patients).

	*n* (%)
Hypertension	165 (54.1)
Diabetes mellitus	85 (27.9)
Solid malignancy	85 (27.9)
Ischemic heart disease and/or valve replacement	44 (14.4)
Hematological malignancy	35 (11.5)
Obesity	33 (10.8)
Renal failure	29 (9.5)
Chronic Obstructive Pulmonary Disease (COPD)	28 (9.2)
Peritonitis	25 (8.2)
Asthma	16 (5.2)
Cirrhosis	14 (4.6)
Pancreatitis	13 (4.3)
Rheumatic disease	11 (3.6)
Kidney transplant	9 (3.0)
Psoriasis	9 (3.0)
HIV/AIDS	8 (2.6)
Crohn’s disease	7 (2.3)

**Table 3:** Distribution of the main causes of immunosuppression among patients with candidemia at Hospital Universitario Central de Asturias (HUCA), Oviedo, Spain, during the study period 2015–2024 (*n* = 305 patients).

	*n*	Total %	% Immunosuppressed
Corticosteroids	124	40.7.	70.1
Immunosuppressant	32	10.5	18.1
Cirrhosis	15	4.9	8.5
Neutropenia	14	4.6	7.9
Severe malnutrition	10	3.3	5.6
Allogenic hematopoietic stem-cell transplantation (allo-HSCT)	9	3.0	5.1
HIV/AIDS	8	2.6	4.5
Aplasia	14	4.6	2.6
Pancytopenia	2	0.7	1.1
Prematurity	2	0.7	1.1
Autologous hematopoietic stem-cell transplantation (AHSCT)	1	0.3	0.6

Footnote: Total % represents the percentage of the overall cohort (*n* = 305 patients), whereas % immunosuppressed represents the percentage among immunosuppressed patients only (*n* = 177).

**Table 4:** Temporal changes in the prevalence of selected predisposing factors for candidemia at Hospital Universitario Central de Asturias (HUCA), Oviedo, Spain, comparing the 2015–2019 (*n* = 124 episodes) and 2020–2024 (*n* = 182 episodes) study periods.

Risk Factors	2015–2019 (*n* = 124)	2020–2024 (*n* = 182)	*p*-Value
Central venous catheter	88 (71.0%)	135 (74.2%)	0.601
Urinary catheter	15 (12.1%)	62 (34.1%)	<0.001
Abdominal surgery	31 (25.0%)	19 (10.4%)	<0.001
Abdominal drainage	26 (21.0%)	19 (10.4%)	0.013
Mechanical ventilation	2 (1.6%)	24 (13.2%)	<0.001
Parenteral nutrition	5 (4.0%)	16 (8.8%)	0.166
Immunosuppression (any)	58 (46.8%)	119 (65.4%)	0.001

Data are presented as *n* (%). Comparisons were performed between patients diagnosed during the pre-pandemic period (2015–2019) and those diagnosed during the pandemic/post-pandemic period (2020–2024).

## Data Availability

The original contributions presented in this study are included in the article. Further inquiries can be directed to the corresponding authors upon reasonable request. The data are not publicly available due to privacy and ethical restrictions.

## Abbreviations

The following abbreviations are used in this manuscript:

ASM	American Society for Microbiology
CFU	Colony-forming unit
CIBER of Respiratory Diseases	(CIBERES)
COVID	Coronavirus Disease
CLSI	Clinical and Laboratory Standard Institute
CRF	Case Report Form
ECCM	European Confederation of medical Mycology
HUCA	Central University Hospital of Asturias
ICU	Intensive care unit
ISCIII	Carlos III Health Institute
ISHAM	International Society for Human and Animal Mycology
ISPA	Health Research Institute of the Principality of Asturias
MALDI-TOF	Matrix-assisted laser desorption ionization-time of -light
PCR	Polymerase chain reaction
ASP	Multidisciplinary Antimicrobial Stewardship Program
